# Smart Consumer Wearables as Digital Diagnostic Tools: A Review

**DOI:** 10.3390/diagnostics12092110

**Published:** 2022-08-31

**Authors:** Shweta Chakrabarti, Nupur Biswas, Lawrence D. Jones, Santosh Kesari, Shashaanka Ashili

**Affiliations:** 1 Rhenix Lifesciences, Hyderabad 500038, India; 2 CureScience, San Diego, CA 92121, USA; 3 Department of Translational Neurosciences, Pacific Neuroscience Institute and Saint John’s Cancer Institute at Providence Saint John’s Health Center, Santa Monica, CA 90404, USA

**Keywords:** digital diagnostics, digital health, wearables, machine learning, personalized healthcare

## Abstract

The increasing usage of smart wearable devices has made an impact not only on the lifestyle of the users, but also on biological research and personalized healthcare services. These devices, which carry different types of sensors, have emerged as personalized digital diagnostic tools. Data from such devices have enabled the prediction and detection of various physiological as well as psychological conditions and diseases. In this review, we have focused on the diagnostic applications of wrist-worn wearables to detect multiple diseases such as cardiovascular diseases, neurological disorders, fatty liver diseases, and metabolic disorders, including diabetes, sleep quality, and psychological illnesses. The fruitful usage of wearables requires fast and insightful data analysis, which is feasible through machine learning. In this review, we have also discussed various machine-learning applications and outcomes for wearable data analyses. Finally, we have discussed the current challenges with wearable usage and data, and the future perspectives of wearable devices as diagnostic tools for research and personalized healthcare domains.

## 1. Introduction

Wearables, which refer to smart consumer devices that record digital health data, are becoming an integral part of our daily lives. This reflects the growing health consciousness among people. Wearable biosensors are low-price, non-invasive, and non-irritating devices that function by continuously measuring a person’s physiological parameters in real time [1,2], which can be used for the early as well as in-depth diagnosis of several conditions. They also facilitate personalized patient health monitoring outside the clinical setting, which is an advantage considering the restricted movements during the COVID-19 pandemic [3,4]. More than 500 health-related sensors are available in the market [1,2,3,4,5], and the sale of such devices has experienced more than a 20% annual growth rate, with an estimated market size of more than EUR 150 billion by 2028 [6]. Wearable devices are available in different forms that are in contact with different body parts, and are also available as devices attached with fabrics. Based on their point of contact, they can be categorized into head, limb (which includes arms), leg, eye, and torso wearable devices [7]. Based on their probing method, they can also be categorized as skin-based or biofluid-based [8]. Apart from consumer devices, wearable devices are available for specialized monitoring, such as wearable smart insoles for diabetic foot monitoring, devices for real-time heart attack detection, and smart-digital stethoscope systems [9], the use of which is often suggested by clinicians. In this review, we have primarily focused on skin-based wrist-wearable consumer devices that provide continuous data, which are used for the diagnosis of several disease conditions.

Wearable devices contain different types of sensors that collect data on step counts, heart rate, sleep duration, calories burnt, stress, and oxygen levels [10]. The parameters measured, collected, and stored by the device could be helpful in the identification of health conditions by detecting deviations from the corresponding baseline values. This technology can also be used to track and connect the user’s daily activities, such as cycling, running, biking, and walking, in combination with GPS, thus providing the location of the activity as well. These data can be affected by other external or environmental factors, such as temperature, altitude, and humidity, imposing additional dimensions on the data. These data are transferred to a cloud-based storage and are accessible by users’ mobile devices as well as to researchers and clinicians for precision diagnostics. To analyze this massive amount of multivariate time-series data, the conventional statistical approach is often inadequate, specifically in the context of making a diagnosis from the unseen data. In this case, machine-learning (ML) algorithms are beneficial and are being used to predict health events, intervention, and prevention [11,12]. In this review, we discuss the use of various ML algorithms for the analysis of data provided by wearable devices. 

Recent research highlights that wearable devices can work as digital diagnostic tools due to their usefulness in detecting several diseases. The remotely accessed, real-time-monitored, continuous data recording in a personalized manner has made wearables an effective tool for the diagnosis of physiological conditions. It has been found that the physiological parameters obtained from these biosensors can be used in the detection of Lyme disease [1], respiratory infections, cardiovascular disorders [13,14], neurological disorders [15], coronavirus diseases [16,17,18], Parkinson’s disease [19], diabetes, liver diseases, and others. Not only can they detect physiological diseases, but wearables can also be employed to diagnose psychological states. This review discusses both the physiological and psychological disorders diagnosed by consumer wearables. For reporting a generalized review, we searched the PubMed database in the month of July 2022 using different combinations of the following keywords: ‘wearables’, ‘consumer wearables’, ‘diagnostics’, ‘smart watch’, and ‘digital health’. The search results showed different types of articles, among which we focused on research articles addressing cardiovascular diseases, neurological diseases, fatty liver diseases, metabolic disorders, sleep disorders, corona virus diseases, and psychological illnesses. Among these, we centered our attention on smart wrist-worn consumer devices. The detailed search method is provided as supplementary information.

The technology that enables the provision of real-time and accurate physiological data with the help of these wearable biosensors is having and will have a broader impact on our daily lives in the future. The use of wearables as diagnostic tools is associated with several difficulties, including achieving and maintaining precision, power consumption, and connectivity. Difficulties are associated with their fabrication, as they require miniaturization and the integration of various sensors [20]. Compared to the conventional lab-based diagnostic methods, these remotely monitored personalized diagnostic methods raise concerns over data security, sharing, and storage [21]. Regulatory bodies play an important role in this landscape. Here in this review, we focused entirely on consumer wrist-wearable devices that provide continuous data; we discuss their usefulness as digital biomarkers for the diagnosis of several diseases, we examine various ML algorithms used for wearable data analysis, and we further elaborate on the challenges associated with wearable technology and their future perspectives. 

## 2. Wearables as Digital Diagnostics 

Wearable devices are revolutionizing the healthcare system by monitoring health even outside the clinics. This has enabled medical practitioners to adapt to wearables for monitoring as well as diagnosing their patients. Here, we discuss the major outcomes obtained from wearable data that are used for digital diagnostics only. Table 1 summarizes different wearable devices and their applications as digital diagnostic tools, as reported in different studies. 

### 2.1. Cardiovascular Diseases

As the primary data generated by wearable devices include the heartbeat rate, step count, and energy consumed, researchers have concentrated on associating cardiovascular disorders with these data. Cardiovascular diseases cause millions of deaths globally every year [46]. Continuous monitoring and the diagnosis of abnormalities are important for reducing fatalities. Wearable technology has made this more feasible [47]. A clinical trial with over 60 adults showed that wearing smartwatches with blood-pressure-monitoring features lowered the patients’ blood pressure and resting heart rate, elucidating the effect of self-monitoring [48]. Self-monitoring can also lead to early diagnosis [49]. In a study by Rens et al., cardiovascular disease patients were made to take a 6-minute walk test (6MWT) and their activity data were collected with an iPhone and an Apple Watch using the VascTrac app. The home-based 6MWT assessed frailty with 83% sensitivity and 60% specificity. Hence, functional capacity and frailty could be monitored in cardiovascular patients safely and with a higher resolution by using wearable devices [22]. Another study by Teo et al. tracked sleep and collected multi-modal phenotypic data and questionnaire responses from normal volunteers. The sleep data derived from the wearables and by self-reporting were compared on the basis of total sleep time (TST) and sleep efficiency (SE). From a data analysis of a multi-modal phenotype, it was found that the TST and SE derived from wearables showed an association with the markers of cardiovascular disease, such as waist circumference and body mass index. However, the self-reported data did not show such associations. A lack of sleep could lead to telomere shortening, which is a tumor suppressor mechanism (premature telomere attrition) (confidence interval [CI] = 74.573–636.538, *p*  =  0.016); hence, the sleep data from wearables were useful for providing insights into the cardiovascular disease risk (β  =  1.275, CI  =  0.187–2.363, *p*  =  0.023) [29]. The usage of wearables has allowed people to track their own heart rhythms for a very long period [50]. By using heart rate and step count data from wearable smartwatches, machine-learning algorithms have been developed by different research groups for detecting atrial fibrillation (AF), which is a leading cause of stroke worldwide. A study by Tison et al. presented a deep-learning algorithm for the detection of AF. The neural network showed a 95% CI of 0.94–1.00 (*p*  <  0.001) for the detection of AF compared to the AF diagnosis based on ECG results, which was used as reference. The sensitivity was observed to be 98%, with 90.2% specificity [23]. Similarly, another study by Inui et al. used wearables such as an Apple Watch and a FitBit and compared them with ECG data for the detection of paroxysmal AF. The correlation between the Apple Watch pulse rate data and the ECG heart rate data was found to be better than that between the FitBit data and the ECG data. The coefficient of determination for the Apple Watch was R^2^ = 0.685, whereas that for the FitBit was R^2^ = 0.057. Hence, the Apple Watch was proven to have better AF detection precision than the FitBit [51]. When the PPG screening app was used for AF detection, a positive predictive value of 91.6% was observed in patients who were confirmed to have AF (CI: 91.5–91.8%) [27]. Bashar et al. also proposed a method of AF detection that detects noise artifacts and motion by performing a time-frequency PPG signal analysis. Further, their algorithm to detect premature atrial contraction was used for AF detection with a higher accuracy. The proposed method showed a specificity, sensitivity, and accuracy of 97.43%, 98.18%, and 97.54%, respectively [28]. In a study by Koshy et al., the researchers monitored sinus rhythm using two different wearables (FitBit and Apple Watch) that collected heart rate data. For the detection of atrial arrhythmias, both the devices showed good results. However, the Apple Watch (*r* = 0.83) showed a better correlation than the FitBit (*r* = 0.56) [52]. Photoplethysmogram (PPG) signals derived from wearables or smartphones could be useful for monitoring cardiac health after signal corruptions and noise are removed. It was found that these denoised PPG signals could effectively predict coronary artery disease (CAD) [53]. Apart from the commercial smartwatches, smartwatches such as Kick LL are being developed for the purpose of monitoring respiration and heart rate [26]. 

Smartwatches have emerged as a new-age diagnostic tool for recording multichannel ECGs [24]. For this purpose, smartwatches can be attached to different body parts such as the chest or abdomen. Samol et al. have shown the possibility of an early ECG differential diagnosis of cardiac diseases [54]. The QT interval was also measured using a smartwatch, and the result showed a correlation of up to 0.994 with standard ECG data [25]. 

### 2.2. Neurological Disorders and Stress

Wearable devices have allowed for the continuous monitoring of our physiology, which has made the detection and treatment of chronic diseases, such as neurological disorders and mental health problems, possible. Electrodermal activity (EDA) shows the activity of the sympathetic nervous system, and thus is a potential tool for tracking arousal and autonomic regulation. EDA data are usually collected from the fingertips, wrists, or ankles. It is known that measuring EDA consumes less power than other monitoring methods and is a simple process. There are EDA-measuring wristbands on the market with embedded EDA sensors where the wristbands are made up of electrically conductive fabric [55]. However, EDA values can be affected by various other factors, including the environmental, skin, and room temperatures [56]. These limitations become especially important when an EDA sensor is employed in a wearable device controlled by temperatures [57]. The EDA sensor indicates the activity of eccrine sweat glands, which varies with the psychological state [58]. There is a positive correlation between EDA values and skin temperature (*r* = 0.13, *p* < 0.001). A study was performed to understand the performance of a student in real-time during an exam [59]. It has also been found that EDA measurements from wearable sensors are useful for detecting epileptic seizures. A surge in EDA was detected during an epileptic seizure, which implies a great sympathetic discharge [15,32,60]. Another study showed that wearable sensors could also be used to detect social anxiety in people, and thereby improve the monitoring and treatment of social anxiety. The data used for this purpose were heart rate, EDA, and skin temperature (ST). This study also demonstrated that these sensors could distinguish among different levels of anxiety in an individual [61]. 

Wearable devices appear to be a useful tool for characterizing different parameters in different dementia-type diseases such as Parkinson’s disease (PD) and Alzheimer’s disease (AD) [62]. Sensors carried by the wrist-worn device StepWatch are used for the quantitative diagnosis of Parkinson’s disease and multiple sclerosis by counting the strides of the users [35]. Researchers are also using inertial sensors in wearables for the continuous detection of rest tremors and dyskinesia in patients suffering from PD [63,64]. The accelerometers in these watches can differentiate between postural tremors and essential tremors in PD patients by calculating the peak harmonic power and frequency. They accurately provide diagnostic information in terms of postural tremors [65,66]. Sigcha et al. also showed a high correlation (0.969) between measurements of resting tremors using smartwatch data and clinical measurements [37]. Based on tremor measurements using wearable devices, the classification between differential diagnoses and healthy patients reached 86.5% precision [67]. EchoWear, a smartwatch-based speech and voice exercise monitoring system, was implemented to detect voice and speech disorders in PD patients [36]. A framework called SPARK, employing wearable devices and smartphones, was developed for the detection of multiple symptoms associated with PD [68]. The early diagnosis of PD is also possible from activity data during sleep and sleep quality data [33]. Apart from the tremor detection, smartwatches are used to measure ‘plate-to-mouth’ time during eating, which reflects the intensity of the disease [34]. 

For AD patients, wearables are used as digital biomarkers [69]. They are used for the inference-based diagnosis of behavioral events using inertial motion data [70]. The early diagnosis of mild cognitive impairments (MCIs) is also possible by using wrist-worn wearables [71]. Apart from the diagnosis, consumer-wearable devices have a great usefulness for patient care and the monitoring of elderly AD patients [72]. By implementing specific sensors into wearable devices, Al-Naami et al. developed a smart wearable device for alerting AD patients to fall-down conditions [73]. 

### 2.3. Fatty Liver Diseases

Nonalcoholic fatty liver diseases (NAFLDs) are rapidly increasing in number and becoming the primary cause of most liver-associated deaths globally. The major cause of all liver diseases is physical inactivity. Wearable devices help individuals to track their physical activity at a minute level. Hence, data from wearable devices act as a wellness indicator for patients suffering with liver diseases. An improvement in physical activity leads to an improvement in cardiorespiratory fitness, and this can be measured with cardiopulmonary exercise testing (CPET). CPET is found to be useful in identifying risks in transplant hepatology [74]. These wearables are not only useful for detecting and identifying liver diseases, but are also useful for keeping track of physical activities that have shown to be helpful for NAFLD and hepatocellular carcinoma (HCC) patients. In a study by Kim et al., patients were monitored using Neofit (Partron Co), which recorded the calories burnt, step count, exercise duration, and heart rate. After 12 weeks of following the exercise program, the body composition and physical fitness significantly improved in the HCC patients who completed their therapy [39]. Similarly, a study by Schneider et al. recorded the physical activity of participants using a wrist accelerometer and detected that an increase in physical activity resulted in a dose-dependent reduction in liver disease, which appeared to be independent of adiposity [38]. 

### 2.4. Corona Virus Diseases

In the context of the pandemic caused by the 2019 coronavirus disease (COVID-19), researchers used data on heart rate, step count, and calories burnt, recorded by wearable devices, to detect COVID-19 infections in pre-symptomatic and asymptomatic conditions [75]. Lonini et al. have demonstrated how these consumer-grade wearables collecting data for a very long period could be useful for detecting the symptoms of such viral infections in an individual. A wearable designed to be worn on the suprasternal notch can track physical activity, cough sounds, and cardio-respiratory function [76]. Snyder et al. used the resting heart rate difference (RHR-diff) method and the heart-rate-over-steps anomaly detection (HROS-AD) method for the early detection of anomalies in the recorded data of COVID-19 patients, even 3 days (median value) before the onset of symptoms [16,17]. In another study, a gradient-boosting algorithm was used to detect an infection and the important symptoms [77]. Quer et al. provided a wearable device data model that complemented conventional virus-testing methods to detect COVID-19 infections [78]. In another study, Bogu and Snyder showed that using wearable data 7 days prior to COVID-19 detection and 21 days after the detection could recognize COVID-19 infections using a deep-learning-based method of a long short-term memory network-based autoencoder (LAAD). LAAD detects COVID-19 based on an abnormal resting heart rate during the period of infection. It was able to detect COVID-19 in the pre-symptomatic period as well as the symptomatic phase of the patients, with a precision score of 0.91 (CI: 0.854–0.967) [10]. Cho et al. proposed a one-class SVM method that can detect COVID-19 23.5–40% earlier compared to the method of Mishra et al. [16,17,18,19,20,21,22,23,24,25,26,27,28,29,30,31,32,33,34,35,36,37,38,39,40,41,42,43,44,45,46,47,48,49,50,51,52,53,54,55,56,57,58,59,60,61,62,63,64,65,66,67,68,69,70,71,72,73,74,75,76,77,78,79].

### 2.5. Metabolic Disorders

Metabolic diseases, such as diabetes, affect millions of people around the world every year. They increase the chance of multiple organ failure and result in a decreased quality of life [80]. Consumer wearables such as fitness trackers are also useful in diabetes patients. It has been found that physical activity (PA) has a major effect on glucose concentration. The effect of PA depends on the intensity, mode, and duration of the exercise [81]. Wearable smart devices are useful tools for the self-monitoring of activity by the patient and for remote monitoring by the caregivers. A clinical trial is ongoing to explore the efficacy of integrated do-it-yourself smartwatch glucose monitoring compared to scanned continuous glucose-monitoring systems [82]. In another study, Fitbit^®^ data from diabetic patients were used to correlate the association of physical activity with glycemic exposure. Further, assessing PA quantitatively may show to be useful in making mealtime treatment decisions. It was also observed that participating in PA every day demonstrated an immediate or later impact on glucose control [30]. Akyol et al. reported a novel consumer-wearable device called Diafit that works as a customizable glucose monitor for diabetes patients [40]. In a study by Weatherall et al., the researchers demonstrated an association between sleep and PA data from these wearable devices (Fitbit Charge HR) and the information reported by the type 2 diabetes mellitus (T2DM) patients themselves. It was observed that the self-reported data were positively associated with both the PA data (*r* = 0.35, *p* = 0.001) and the sleep data (*r* = 0.24, *p* = 0.04) [31]. Hence, it is believed that monitoring patients extensively could allow them to make decisions on disease treatments. In addition, data from these wearables have the potential to improve patient-reported outcomes and their care. There is also a non-invasive method of monitoring glucose that is performed by pressing the wrist or fingertip on the thin glass behind any smart wristwatch, which consist of a chemochromic mixture that has the same function as a PPG sensor. These chemochromic components facilitate the measurement of various metabolites from sweat, which are further used to obtain the glucose concentration using neural network algorithms built into the PPG sensor. The values obtained from this showed a high correlation with invasive methods of monitoring glucose. Hence, wearables provide a non-invasive, miniaturized, easy-to-operate, and novel method for glucometry, which could be used as an alternative to invasive tools in clinical settings [83]. In another study reported by Lee et al., smartwatch data along with other digital data were used to enable the better prevention of metabolic syndromes by the continuous detection of several health factors [41]. 

### 2.6. Sleep Quality

Sleep is important for normal bodily functions and for good health. A lack of sleep can have physical, emotional, and mental effects and can lead to serious health conditions, especially among diseased individuals. Both PA and sleep are related to each other. Wearable technology is currently being used to track PA and sleep, which could help researchers study sleep science in-depth, resulting in the better diagnosis of sleep-related disorders [84]. Sathyanarayana et al. demonstrated that deep learning can be used to predict sleep quality (whether it was good or poor) by making use of an actigraph obtained from the waking hours of an individual [85]. In another study by Berryhill et al., it was reported that a wearable sleep tracker could improve sleep quality in healthy people and track the quality as well as quantity of sleep. They also compared the sleep quality measured by wearables and polysomnography. The wearables showed a low precision error (17.8 min) when measuring sleep duration [44]. Currently, there are so many sleep trackers available on the market that it is difficult to discern which one is the best. Lees et al. performed a comparison among various wearables that track sleep and time in bed by using a sleep diary (SD). The Jawbone UP3 and Fitbit Charge Heart Rate devices showed the greatest equivalence to the SD in terms of sleeping time. The SenseWear Armband, Garmin Vivosmart, and Jawbone UP3 devices showed the greatest equivalence to the SD in terms of time in bed [86]. Meharabadi et al. used a wearable ring and watch to measure sleep quality, and observed that for total sleep time, the correlation of the actigraphy data with the ring data was 0.86 (*p* < 0.001); with the watch data, the correlation was much lower, at 0.59 (*p* < 0.001) [42]. Topalidis et al. also observed that wrist-worn device data and actigraph reports that derived the wake-up time and sleep time had high correlations (0.96 and 0.84, respectively; *p* < 0.001) with subjective reports [87]. In a study conducted by Chen at al., a PPG smartwatch outperformed the polysomnography method for detecting obstructive sleep apnea. An accuracy of 81.1% was achieved [43]. Papini et al. also observed that a wrist-worn PPG-integrated smartwatch could complement the standard apnea diagnostic techniques with a relatively lower correlation of 0.61 [88]. Ko et al. conducted a study on sleep quantification in PD patients using smartwatches, and detected abnormal rapid eye movements. They also observed that the percentage of the deep-sleep stage differs between healthy (38.1) and PD (22.0) patients [89].

### 2.7. Psychological Illness

Apart from detecting physiological illnesses, wearable devices play an important role in addressing psychological characteristics that are often neglected due to a lack of symptomatic evidence. Wearable device data equipped with ML algorithms are helpful for extracting the highly personalized nature of psychological conditions such as depression and mood swings. A recent study on 14 young people using EEG data, neurocognitive assessments, and lifestyle data from wearable devices revealed that each person had distinct depression determinants [90]. Hence, highly personalized diagnoses and treatments are required. In another interesting study, pictures were shown to the participants of the study. A machine-learning analysis further identified important features, and classifiers were used to predict the valence and arousal. Although the accuracy was not significantly high (69.9%), it showed the possibility of identifying emotional states using wearable devices [91,92,93]. Apart from the emotional state, the supervised machine-learning and gradient-boosting algorithm DART (dropouts meet multiple additive regression trees) [94] has been used for the detection of depression in a group of working young people wearing a Fitbit wristband. This was further evaluated by performing a k-fold cross-validation on the test sets. The study showed that the severity of depression symptoms was associated with nighttime heart rate variation [45]. Anxiety and depression have also been diagnosed in children with the help of wearable data devices and a machine-learning method such as k-nearest neighbor (kNN). A diagnosis accuracy of 75% was achieved by using the kNN method [95]. Stress is another mental health issue that has become very prevalent among adults. A study by Nath and Thapliyal proposed a new prototype for detecting stress among people using a wristband embedded with EDA, PPG, and ST sensors, which provided EDA, blood volume pulse (BVP), IBI, and ST signals that could distinguish between the stressed and non-stressed state of a person [96].

## 3. Role of Machine Learning in Diagnostics

**Figure 1:**
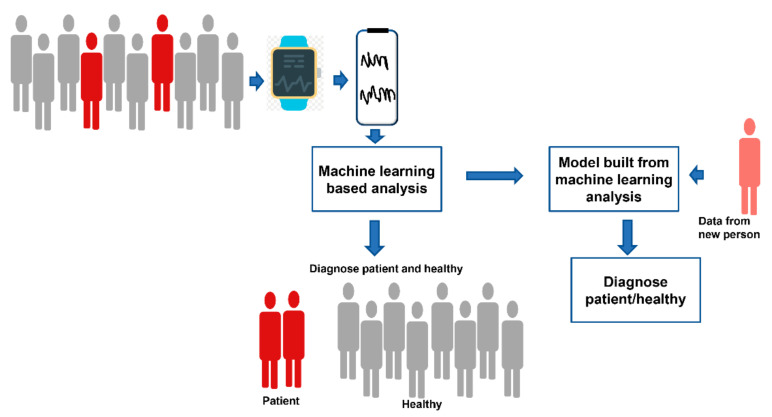
Schematic diagram showing how machine learning algorithms are used for the analysis of data extracted from wearable devices.

Very often, the data from wearable devices are used as an additive to other medical data. These wearable devices generate a huge amount of multivariate time-series data. Extracting deeper insight from the first level of data requires data preprocessing and extensive analyses, for which machine-learning (ML) algorithms have become an indispensable tool for researchers [97]. Machine learning belongs to the field of artificial intelligence. In ML, programs perform some tasks, learn from the performance, and perform new tasks based on their prior learning. Figure 1 shows how machine-learning algorithms are used for the analysis of data extracted from wearable devices. 

In the context of big data, ML-based algorithms have outperformed conventional algorithms. Moreover, wearable devices are particularly useful for revealing the underlying personalized characteristics of several physiological and psychological diseases. Providing personalized diagnoses and treatments has become feasible due to ML algorithms. In this section, we discuss different ML algorithms used for the analysis of data from wearable devices and their outcomes in the context of different diseases. 

An analysis of wearable device data is primarily focused on identifying anomalous behaviors in the recorded data, and also predicting future events [98]. This is achieved by training the machine-learning model with the recorded data of known anomalous events and testing the model’s performance with previously unseen data. Apart from statistical methods based on the resting heart rate difference coupled with step counts [16,17], ML algorithms such as the support vector machine (SVM) method [99], the random forest (RF) method [100], gradient boosting decision trees [101], and the k-nearest neighbors (kNN) method [102] have been used. Among them, SVM performs best [103]. These conventional algorithms were used to build models for analyzing ECG data, and these models were used for stress classification based on smartwatch data. Feature engineering played an important role in improving the performance of the built models [104]. Feature engineering includes converting time-series data to the frequency space and extracting seasonality, frequency spectra, and power spectral density [105]. 

Machine-learning algorithms have been applied to data from implanted electroencephalography (EEG) electrodes and wearable devices for the detection of epileptic seizures, as well as for the prediction of seizure events. The wearable devices have a reported sensitivity of more than 90% for detecting seizures [106]. Weiting et al. used several algorithms, including SVM, RF, and naive Bayes, to build an ML algorithm ensemble for the purpose of predicting the cardiovascular risk from wearable healthcare data-collection devices [107]. In a study involving 407 participants using smartwatches, a gradient-boosting algorithm identified and predicted SARS-CoV2 infections [108]. Researchers have used multiple-instance learning via an embedded instance selection (MILES) method for feature transformation to detect obstructive cardiomyopathy [109]. However, conventional algorithms such as kNN perform better than deep-learning approaches at detecting out-of-distribution events for human activity recognition [110]. Neural networks are the building blocks of deep-learning methods. The autoregressive integrated moving average (ARIMA) model [111], which evolved from neural networks, is popularly used for time-series data analyses. It has also been used to analyze data from wearables. The ARIMA model is a type of auto-regression model. It predicts future observations based on past observations, while considering seasonal effects. The current value of time-series data is considered as the linear combination of past records. Applying random forest and ARIMA on blood pressure data has identified a personalized dependence of blood pressure on other lifestyle factors [112]. DeepBeat, a deep-learning method based on a convolution neural network (CNN), has been developed to assess data quality as well as abnormal heart rhythms and atrial fibrillation [14]. In a recurrent neural network (RNN), the features are connected by temporal sequences and the past inputs are stored for a certain amount of time, which often leads to vanishing gradient problems that can be overcome by long short-term memory (LSTM) algorithms [113,114]. LSTM algorithms are also used for predictive analyses. Matthew et al. used ARIMA and LSTM along with other ML models to predict future heart rate irregularities, and they observed that ARIMA performed better compared to the other algorithms [115]. The LSTM-based method has successfully been used to detect COVID-19 using wearable data [10]. LSTM has also been used for detecting congestive heart failure [116]. Cho et al. claimed that one-class SVM provided a 23.5–40% earlier detection of COVID-19 compared to the LSTM method [79]. LSTM has also been applied for estimating sleep stages from wearable data [117]. 

In addition, several ML-based algorithms and platforms have been developed for analyzing wearable data. PRISM uses Fourier-transform-based engineered features coupled with text data, analyzed by text mining, to provide a data-driven platform for monitoring mental health [118]. A correlation-based emotion recognition algorithm (CorrNet) recognizes emotions when a person watches videos. It also employs feature engineering based on correlations [119]. Kong et al. developed an algorithm that can remove non-stationary motion artefacts in heart rate data by converting the data into a frequency domain [120]. The ROAMM framework was developed to detect the real-time activity of a user. It is coupled with a server for remote analysis [121]. The deep-learning-based android app ‘SmartFall’ uses smartwatch data to detect falls [122]. Kwon et al. implemented a neural-network-based smartwatch interface for the recognition of gesture patterns [123]. The Roche PD Mobile Application was developed for the remote quantification of motor sign severity in early-stage PD patients [124]. Zylstra et al. developed a mobile health platform for the daily collection of clinically relevant measurements for patients with neurological disorders [125]. The iSenseSleep app works to detect sleep duration based on wearable data and smartphone usage data [126].

## 4. Future Perspectives and Challenges

Advancements in technology have allowed for the generation of wearables that can track data, such as heart rate, steps, and calories, in a humongous amount. Researchers have now started to branch out from physical activity tracking to focusing more on major healthcare challenges, including diabetes management and the remote monitoring of older individuals. Hence, to achieve this goal, researchers have been working on the development of biosensors that are incorporated with bioreceptors such as antibodies, enzymes, or cell receptors [127]. 

The rapid progress in the development of wearables is very evident from the increase in the reporting rate of proof-of-concept studies. However, there are many challenges associated with wearables in healthcare. One of the major challenges of using wearables as smart diagnostic tools is associated with their precision and accuracy. A recent study by Filippo et al. highlighted the deviation in the results obtained by smartphone applications and wearable devices [128]. In addition, the results from different devices vary. Gloria et al. performed a comparative study involving different smartwatches [129]. Scarlet et al. compared the diagnostic accuracy of smartwatches for detecting cardiac arrhythmia [130]. Hahnen et al. conducted a study with over 127 individuals and observed that the accuracy and precision of heart rate data met the accuracy guidelines, but the blood pressure and oxygen saturation data failed the guidelines [131]. In a study conducted by Nelson et al., the data from an Apple Watch and a Fitbit device were compared with ambulatory echocardiogram (ECG) data collected from the same subject. The Apple Watch and Fitbit data showed agreement with the ECG data up to 95% and 91%, respectively [132]. However, the Fitbit data did not outperform the ECG data in the detection of epileptic seizures [133]. The accuracy of wearable devices must at least be at a comparative level with the conventional diagnostic methods. 

Another challenge is the energy consumption, especially in MEMS-based inertial measurement units and wearable sensors. There is a need to reduce the order of magnitude of the energy in sensing and wireless communication by utilizing technologies that can help with energy reduction and overcome this challenge. Most of these technologies are under clinical evaluation and require regulatory approvals before commercialization [134]. To manage the energy consumption, the size of sensors has been reduced. Wearable devices also require internet access. A limited internet connectivity limits the use of wearables in the rural areas of under-developed countries. For poverty-stricken countries, the current cost of wearables and internet service has made wearables out-of-reach for many people. The wearability of these devices is also an issue. Users prefer them to be comfortable and light enough to wear and carry around, without hindering their daily activities. Hence, the tradeoff between the complexities associated with the computations and the weight and size of the wearable is one of the major challenges. Another challenge is the safety of the user, which could come into the picture when using wearable devices that use wireless technology for transferring data and that involve radiation, which could have a negative effect on the user’s health [135]. 

Data security and privacy are other major challenges when it comes to data from wearables. Implementing security policies while maintaining the size and computational complexities of the wearables is quite a challenge. Wearables have poor data encryption and protection. Patients also have concerns over data security and may refuse to use wearable devices [136]. The health literacy of patients is an associated issue. 

The application of wearables comes with many regulations and legal frameworks, as it involves individual data collection, processing, storing, sharing, and further analysis for research purposes. Hence, the privacy and security of an individual’s sensitive information comes into question [137]. Vast applications of wearable technology could be possible, especially through the development of regulatory modifications in the data privacy aspect. It has become important to make the data exchange among health app providers, wearable manufacturers, and health insurers more transparent [138]. These aspects may also create a barrier in the market. Every country has their own requirement or certification policy for market access, and these need to be considered during the early stages of product development. There are different acts that protect and secure the data of every individual. These acts include the Health Insurance Portability and Accountability Act of 1996 (HIPAA) in the USA and the General Data Protection Regulation ((EU) 2016/679, GDPR) in Europe [21]. 

There is an immense scope and necessity for advancements in wearable technology and data processing. These can be achieved by incorporating the Internet of things (IoT), which has numerous applications [135,139,140]. The successful application of wearables as diagnostic tools also involves identifying and addressing the concerns of clinicians, healthcare providers, researchers, industry, and users [141]. 

## 5. Conclusions

The advancements in wearable technology have expanded the horizon of medical research in many directions. Wearable technology has manifested in divergent forms that can be carried on the wrist, head, foot, and other body parts. Among them, wrist-worn devices are most used and do not need any intervention from clinicians [142]. This drove us to set an objective to investigate the current landscape of wrist-worn wearables. We focused on wrist-worn devices working as digital diagnostic tools because of their potential to leverage the healthcare system in different circumstances, such as caring for elderly people, providing remote healthcare, and providing healthcare in ailing socio-economic conditions. Wearables have proven their usefulness in the diagnosis and monitoring of diseases such as cardiovascular diseases, neurological diseases, liver diseases, and even coronavirus diseases. It has also been shown that there are various machine-learning models and algorithms that can be applied for the analysis of wearable data. The growth in this field has led to the early detection of diseases, a faster response to drugs, and higher health literacy, resulting in better patient outcomes. Further enhancements in wearable technology are required to overcome the current challenges as discussed in this paper, including data security and privacy through improved regulation mechanisms. 

## Figures and Tables

**Table 1:** List of consumer-grade wearable devices used as digital diagnostic tools.

Smartwatch Brand	Acquisition Points	Data Collected	Usage	Benefits	References
Apple Watch	Wrist	Step count and heart rate	Monitors frailty in cardiovascular patients when 6MWTs are conducted in both clinical settings and at home.	Assesses frailty with 90% sensitivity in a clinical setting and with 83% sensitivity at home.	[22]
			Detects AF by training using a deep-learning network.	Assesses heart beat rhythm by using a trained deep-learning algorithm with a sensitivity of 98%.	[23]
	Wrist, finger, chest, and abdomen	Heart rate	Could be useful in the detection of several cardiovascular diseases such as myocardial ischemia or cardiac arrhythmias.	Recording from this smartwatch shows feasibility, with a good signal quality of ECG (QT interval) and a correlation of 0.994.	[24,25]
Kick LL	Wrist	Respiration and heart rate	Measures respiration and heart rate using a PPG sensor.	Allows real-time and remote measurements.	[26]
Honor Band 4 and Huawei Watch GT	Wrist	Heart rate	Early AF screening and management with a CI of 91.5–91.8%.	Early detection of AF can prevent strokes or other complications.	[27]
Simband (Samsung)	Wrist	Heart rate	Detects AF using a PPG signal with an accuracy of 98.18%.	Enables easy and non-invasive monitoring of arrythmia.	[28]
Fitbit Charge HR	Wrist	Sleep	Acts as cardiovascular disease and leukocyte telomere length-shortening markers.	Monitors sleep patterns and quality to understand the cardiovascular risk and premature telomere shortening of an individual.	[29]
		Step count and sleep	Tracks physical activity in diabetic patients.	The physical activity record could have an impact on glucose control.	[30,31]
E4 Empatica Wristband	Wrist	EDA and temperature	Uses EDA recordings to monitor the activity of the sympathetic nervous system during epileptic seizures.	Allows continuous and long-term measurements of EDA.	[32]
Huawei Watch 2	Wrist	Sleep	Detects PD at an early state using the sleep patterns of an individual.	Smartwatch-based detection shows a significant correlation of 0.46 to the clinical setting.	[33]
		The 3D acceleration and orientation of velocity signals	Measures movement with inertial sensors in PD patients.	Assesses the eating difficulties in PD patients.	[34]
StepWatch	Wrist	Step count	Step activity monitor (SAM) to count strides; shows a correlation of 0.99 and 1.0 with the gold standard (GaitMait) in PD and MS patients, respectively.	Reliable, easy-to-use, and valid step monitoring tool for PD and MS patients.	[35]
EchoWear	Wrist	Audio	Speech and voice exercise monitoring system for the detection of voice and speech disorders in PD patients.	Remotely monitors the improvements in speech and voice in PD patients.	[36]
Dytran 302M3	Wrist	Tremor constancy and amplitude	Detects tremors in PD patients; shows a strong correlation of 0.969 with the clinical setting.	Provides relevant information about tremors during the early stages of PD and results in improvements in the clinical evaluation.	[37]
Axivity AX3	Wrist	Heart rate, step count, and calories	Tracks physical activities to detect the risk of liver diseases.	Provides a framework for the personalized prevention of liver disease.	[38]
Neofit (Partron Co)	Wrist	Calories burnt, step count, exercise duration, and heart rate	Monitors physical activities in hepatocellular carcinoma patients.	Tracks the activities of patients using the wristband, which correlates with a significant improvement in their health.	[39]
Fitbit, Apple Watch, Garmin, and others	Wrist	Heart rate, calories burnt, step count, and sleep duration	Detects COVID-19 illness	Detects COVID-19 illness in a pre-symptomatic condition.	[16]
Diafit	Wrist, finger, and ear	Glucose	Monitors glucose for diabetic patients.	Consists of various modular accessories required for the assembling of customizable glucose monitors.	[40]
Galaxy Watch Active 1	Wrist	Calories burnt, step count, exercise duration, and heart rate	Manages metabolic syndrome risks by tracking physical activities.	The tracking of physical activities using the smartwatch results in a reduction in waist circumference, blood pressure, and blood sugar by 40%.	[41]
Samsung Gear Sport Watch	Wrist	Sleep	Assesses sleep quality by evaluating sleep parameters; shows a significant correlation of 0.59 with an actigraphy report.	Enables long-term home-based sleep monitoring.	[42]
GT2 (Huawei)	Wrist	Sleep	Used in the screening of obstructive sleep apnea.	Compared to other sleep apnea tests, the smartwatch-based test outperformed the others with an accuracy of 87.9%.	[43]
WHOOP, Inc.	Wrist	Sleep	Tracks sleep with a low bias of 13.8 min and precision errors of 17.8 min.	Accurately measures both dream and slow-wave sleep.	[44]
FitBit Charge 2	Wrist	Steps, heart rate, energy expenditure, and sleep	Tracks physical activity and sleep to understand the behavior and physiology to detect mental disorders such as depression.	A supervised machine-learning algorithm with these data was able to detect the risk of depression with an accuracy of 80%.	[45]

## Data Availability

Not applicable.

## Supplementary Materials

The following supporting information can be downloaded at: https://www.mdpi.com/article/10.3390/diagnostics12092110/s1, Methods for selecting research articles.
