# Supplementary material for: Smart Consumer Wearables as Digital Diagnostic Tools: A Review
Source: Diagnostics (Basel). 2022 Aug 31;12(9):2110. doi: 10.3390/diagnostics12092110 (PMC9498278; doi:10.3390/diagnostics12092110)
Supplement: Supplementary file 1 [file diagnostics-12-02110-s001.zip › diagnostics-1862778-supplementary.pdf]

Supplementary Information

Review

Smart Consumer Wearables as Digital Diagnostics Tools: A Review

Shweta Chakrabarti[1], Nupur Biswas[1*], Lawrence D. Jones[2], Santosh Kesari[3] and Shashaanka Ashili[2]

1 Rhenix Lifesciences, Hyderabad 500038, India
2 CureScience, San Diego, CA 92121, U.S.A
3 Department of Translational Neurosciences, Pacific Neuroscience Institute and Saint John's Cancer Institute at Providence Saint John's Health Center, Santa Monica, CA 90404, U.S.A.

* Correspondence: nupur@rhenix.org

Methods for selecting research articles:

For exploring the role of smart consumer wearables as diagnostics tools we searched PubMed database in English language during the period of 1st to 3rd July 2022. We used different keywords, 'wearables', 'consumer wearables', 'diagnostics', 'smart watch', and 'digital health'. We have also used different combinations of keywords, 'wearables and diagnostics', 'smart watch and diagnostics', wearables and digital health, 'consumer wearables and diagnostics'. All search were held for 'All fields'. Search results were saved in 'Abstract (text)' format. Within the search results PubMed offers filter for different article types. As there is no filter based on research article only, the selection was carried out manually. The selection process, i.e. inclusion/exclusion of any research article in our review was done primarily based on the abstracts. For any article, if we were not able to decide on selection from the abstract, we read the article and finalized our decision. The first step was focused on research articles addressing cardiovascular, neurological, fatty liver, metabolic disorder, sleep disorder, corona virus diseases, and psychological illness. In the second step of selection process, we selected articles where wrist worn smart consumer wearables were used. In the third and final step, we went through the articles and chose articles based on (1) whether the study used wearables as diagnostics tools, and (2) the study shows future potential of use of wearables as diagnostics tools. This evaluation was done based on our own assessment of the literature.
